# 4-Nitro­benzoic acid–*N*-(pyrimidin-2-yl)aniline (1/1)

**DOI:** 10.1107/S1600536811044175

**Published:** 2011-10-29

**Authors:** Aina Mardia Akhmad Aznan, Zanariah Abdullah, Seik Weng Ng, Edward R. T. Tiekink

**Affiliations:** a Department of Chemistry, University of Malaya, 50603 Kuala Lumpur, Malaysia; b Chemistry Department, Faculty of, Science, King Abdulaziz University, PO Box 80203 Jeddah, Saudi Arabia

## Abstract

Four independent mol­ecules comprise the asymmetric unit of the title co-crystal, C_10_H_9_N_3_·C_7_H_5_NO_4_, two for each component. Small conformational differences are noted for the benzoic acid derivatives, notably in the twists of the carb­oxy­lic acid residue out of the plane of the benzene ring to which it is connected [torsion angles = 167.62 (17) and 174.54 (17)°]. In the aniline derivative, the major difference is observed in the dihedral angles formed between the CN_3_ and phenyl least-squares planes [1.51 (5) and 6.25 (6)°]. Pairs of mol­ecules associate *via* O—H⋯N and N—H⋯O hydrogen bonds leading to eight-membered {⋯HOCO⋯HNCN} hetero-synthons. The two-mol­ecule aggregates are consolidated in the crystal structure by C—H⋯O(nitro) and π–π inter­actions [shortest centroid–centroid distance between benzene rings = 3.6242 (10) Å].

## Related literature

For related studies in co-crystal formation, see: Wardell & Tiekink (2011 ▶). For the structure of *N*-(pyrimidin-2-yl)aniline, see: Badaruddin *et al.* (2009 ▶). For the structure of 4-nitro­benzoic acid, see: Tonogaki *et al.* (1993 ▶).
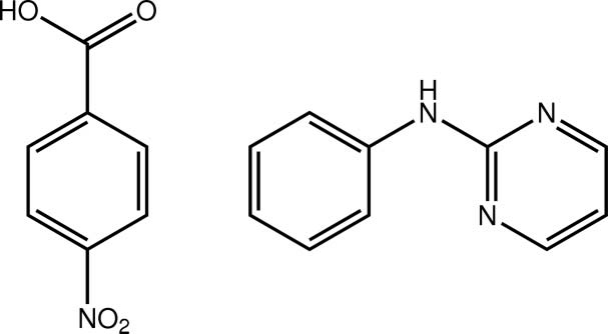


## Experimental

### 

#### Crystal data

C_10_H_9_N_3_·C_7_H_5_NO_4_
*M* *_r_* = 338.32
Monoclinic, 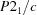

*a* = 12.7754 (4) Å
*b* = 25.7788 (8) Å
*c* = 9.5813 (3) Å
β = 104.209 (4)°
*V* = 3058.92 (17) Å^3^
*Z* = 8
Mo *K*α radiation
μ = 0.11 mm^−1^
*T* = 100 K
0.35 × 0.30 × 0.25 mm

#### Data collection

Agilent SuperNova Dual diffractometer with an Atlas detector
Absorption correction: multi-scan (*CrysAlis PRO*; Agilent, 2010 ▶) *T*_min_ = 0.856, *T*_max_ = 1.000
15808 measured reflections
6818 independent reflections
5093 reflections with *I* > 2σ(*I*)
*R*_int_ = 0.031

#### Refinement

*R*[*F* ^2^ > 2σ(*F* ^2^)] = 0.047
*wR*(*F* ^2^) = 0.118
*S* = 1.03
6818 reflections
463 parameters
4 restraints
H atoms treated by a mixture of independent and constrained refinement
Δρ_max_ = 0.26 e Å^−3^
Δρ_min_ = −0.28 e Å^−3^

### 

Data collection: *CrysAlis PRO* (Agilent, 2010 ▶); cell refinement: *CrysAlis PRO*; data reduction: *CrysAlis PRO*; program(s) used to solve structure: *SHELXS97* (Sheldrick, 2008 ▶); program(s) used to refine structure: *SHELXL97* (Sheldrick, 2008 ▶); molecular graphics: *ORTEP-3* (Farrugia, 1997 ▶), *QMol* (Gans & Shalloway, 2001 ▶) and *DIAMOND* (Brandenburg, 2006 ▶); software used to prepare material for publication: *publCIF* (Westrip, 2010 ▶).

## Supplementary Material

Crystal structure: contains datablock(s) global, I. DOI: 10.1107/S1600536811044175/ez2266sup1.cif

Structure factors: contains datablock(s) I. DOI: 10.1107/S1600536811044175/ez2266Isup2.hkl

Supplementary material file. DOI: 10.1107/S1600536811044175/ez2266Isup3.cml

Additional supplementary materials:  crystallographic information; 3D view; checkCIF report

## Figures and Tables

**Table 1:** Hydrogen-bond geometry (Å, °)

*D*—H⋯*A*	*D*—H	H⋯*A*	*D*⋯*A*	*D*—H⋯*A*
O2—H2o⋯N2	0.87 (1)	1.70 (1)	2.5581 (18)	171 (2)
O6—H6o⋯N5	0.85 (1)	1.78 (1)	2.6230 (18)	171 (2)
N1—H1n⋯O1	0.88 (1)	2.15 (1)	3.0217 (18)	176 (2)
N4—H4n⋯O5	0.88 (1)	2.14 (1)	3.0190 (19)	180 (2)
C2—H2⋯O8	0.95	2.41	3.201 (2)	141
C12—H12⋯O4	0.95	2.43	3.106 (2)	128
C18—H18⋯O7^i^	0.95	2.56	3.330 (2)	138
C19—H19⋯O3^ii^	0.95	2.60	3.518 (2)	163
C31—H31⋯O8^iii^	0.95	2.55	3.238 (2)	129

Symmetry codes: (i) 
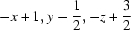
; (ii) 
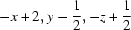
; (iii) 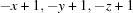.

**Fig. 1.**
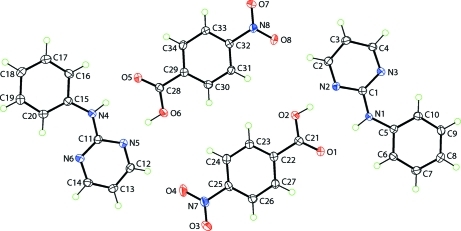
The molecular structures of the four molecules comprising the asymmetric unit in (I) showing the atom-labelling scheme and displacement ellipsoids at the 50% probability level.

**Fig. 2.**
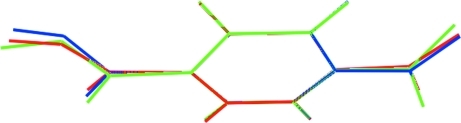
An overlay diagram of the two 4-nitrobenzoic acid molecules in (I) with the literature structure (see text). The red and blue images illustrate the O1- and O5-containing molecules of (I), respectively, and the green image illustrates the literature structure.

**Fig. 3.**
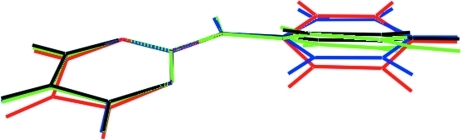
An overlay diagram of the two N-(pyrimidin-2-yl)aniline molecules in (I) with the literature structure which also contains two independent molecules (see text). The red and blue images illustrate the N1- and N4-containing molecules of (I), respectively. The green and black images illustrate the two molecules in the literature structure.

**Fig. 4.**
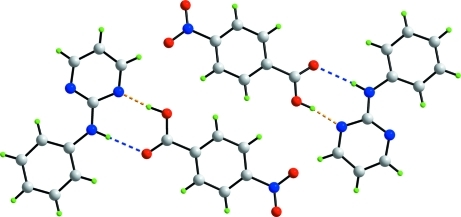
Two supramolecular two molecule aggregates in (I) mediated by O—H···N and N—H···O hydrogen bonds shown as orange and blue dashed lines, respectively.

**Fig. 5.**
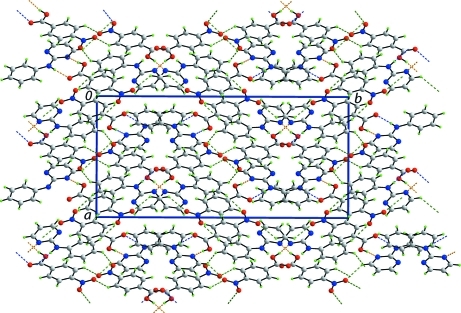
A view in projection down the c axis of the unit-cell contents of (I). The O—H···N, N—H···O and C—H···O interactions are shown as orange, blue and green dashed lines, respectively.

## supplementary crystallographic information

### Comment

In continuation of recent studies into the phenomenon of co-crystal formation (Wardell & Tiekink, 2011), the title 1:1 carboxylic acid–secondary amine co-crystal, (I), was prepared. There are two independent molecules of each of 4-nitrobenzoic acid and *N*-(pyrimidin-2-yl)aniline comprising the crystallographic asymmetric unit of (I), Fig. 1.

The two independent molecules of 4-nitrobenzoic acid differ from each other marginally, and from the conformation found in the crystal structure of 4-nitrobenzoic acid (Tonogaki *et al.*, 1993), Fig. 2. In each molecule of (I) there are small twists of the carboxylic acid residue out of the plane of the benzene ring to which it is connected, *i.e*. the O1—C21—C22—C23 and O5—C28—C29—C30 torsion angles are 167.62 (17) and 174.54 (17)°, respectively, and deviate from planarity compared with the equivalent torsion angle of 178.39 (4)° found in the literature structure (Tonogaki *et al.*, 1993). Less variation is noted for the relative orientation of the nitro groups in (I). Thus, the O3—N7—C25—C26 and O7—N8—C32—C33 torsion angles of 2.7 (3) and -3.0 (2) °, respectively, indicate a similarity in their orientations in (I), and smaller deviations from co-planarity than observed in the literature structure [torsion angle = -14.78 (7)°].

Differences are also noted in the relative conformations found for the *N*-(pyrimidin-2-yl)aniline molecules in (I) and with those found in the crystal structure of *N*-(pyrimidin-2-yl)aniline (Badaruddin *et al.*, 2009), Fig. 3. For the two independent molecules in (I), one of the pyrazinyl rings is twisted out of the CN_3_ plane [the C4—N3—C1—N1 torsion angle is -174.73 (16)°] whereas the other sees both least-squares planes co-planar; the C14—N6—C11—N4 torsion angle is -0.4 (3)°. In the literature structure, the two planes are effectively co-planar with the greatest deviation in the comparable C—N—C—N torsion angles being 177.12 (10)°. Significantly greater differences are noted in the twists between the CN_3_ and benzene least-squares planes. Thus, in (I), the two dihedral angles indicate a close to co-planar relationship between these residues, *i.e*. 1.51 (5) and 6.25 (6)°. By contrast, in the literature structure of *N*-(pyrimidin-2-yl)aniline (Badaruddin *et al.*, 2009), the equivalent dihedral angles are 31.47 (4) and 29.59 (4)°, *i.e*. indicating significant twisting in the molecules.

In the crystal structure, two pairs of the four independent molecules comprising the asymmetric unit are connected into two molecule aggregates, Table 1 and Fig. 4. Eight-membered hetero-synthons are formed as the each carboxylic acid residue forms hydrogen bonds with the amino-H and one of the pyrazinyl-N atoms. The two molecular aggregates are connected into the three-dimensional architecture *via* C—H···O(nitro) interactions, Table 1, and several π–π interactions. The shortest of the latter, 3.6242 (10) Å, occurs between centrosymmetrically related (C22–C27)^i^ rings; symmetry operation *i*: 2 - *x*, 1 - *y*, 1 - *z*. A view of the unit cell contents is shown in Fig. 5.

### Experimental

*N*-(Pyrimidin-2-yl)aniline (0.05 g, 0.00029 mol) and 4-nitrobenzoic acid (0.048 g, 0.00029 mol) were refluxed in a solution comprising ethyl acetate (3 ml) and tetrahydrofuran (3 ml) for 2.5 h at 333 K. The mixture was then stirred overnight and left for crystallization. Yellow crystals formed after three to four days; *M*.pt: 397–398 K.

### Refinement

The H-atoms were placed in calculated positions (C—H 0.95 Å) and were included in the refinement in the riding model approximation, with *U*_iso_(H) set to 1.2*U*_equiv_(C). The O– and N-bound H-atoms were located in a difference Fourier map but were were refined with distance restraints of 0.84±0.01 (O—H) and 0.88±0.01 Å (N—H), and with *U_iso_*(H) = *yU_eq_*(O or N) with *y* = 1.5 for O and *y* = 1.2 for N. Four reflections, *i.e*. (0 1 1), (6 3 6), (7 7 7) and (9 6 3), were omitted from the final refinement owing to poor agreement.

### Crystal data

**Table:**

C_10_H_9_N_3_·C_7_H_5_NO_4_	*F*(000) = 1408
*M**_r_* = 338.32	*D*_x_ = 1.469 Mg m^−^^3^
Monoclinic, *P*2_1_/*c*	Mo *K*α radiation, λ = 0.71073 Å
Hall symbol: -P 2ybc	Cell parameters from 5286 reflections
*a* = 12.7754 (4) Å	θ = 2.3–29.3°
*b* = 25.7788 (8) Å	µ = 0.11 mm^−^^1^
*c* = 9.5813 (3) Å	*T* = 100 K
β = 104.209 (4)°	Block, colourless
*V* = 3058.92 (17) Å^3^	0.35 × 0.30 × 0.25 mm
*Z* = 8	

### Data collection

**Table:**

Agilent SuperNova Dual diffractometer with an Atlas detector	6818 independent reflections
Radiation source: SuperNova (Mo) X-ray Source	5093 reflections with *I* > 2σ(*I*)
Mirror	*R*_int_ = 0.031
Detector resolution: 10.4041 pixels mm^-1^	θ_max_ = 27.5°, θ_min_ = 2.3°
ω scan	*h* = −16→16
Absorption correction: multi-scan (*CrysAlis PRO*; Agilent, 2010)	*k* = −25→33
*T*_min_ = 0.856, *T*_max_ = 1.000	*l* = −12→11
15808 measured reflections	

### Refinement

**Table:**

Refinement on *F*^2^	Primary atom site location: structure-invariant direct methods
Least-squares matrix: full	Secondary atom site location: difference Fourier map
*R*[*F*^2^ > 2σ(*F*^2^)] = 0.047	Hydrogen site location: inferred from neighbouring sites
*wR*(*F*^2^) = 0.118	H atoms treated by a mixture of independent and constrained refinement
*S* = 1.03	*w* = 1/[σ^2^(*F*_o_^2^) + (0.0435*P*)^2^ + 1.2445*P*] where *P* = (*F*_o_^2^ + 2*F*_c_^2^)/3
6818 reflections	(Δ/σ)_max_ < 0.001
463 parameters	Δρ_max_ = 0.26 e Å^−^^3^
4 restraints	Δρ_min_ = −0.28 e Å^−^^3^

### Special details

**Table:**

Geometry. All s.u.'s (except the s.u. in the dihedral angle between two l.s. planes) are estimated using the full covariance matrix. The cell s.u.'s are taken into account individually in the estimation of s.u.'s in distances, angles and torsion angles; correlations between s.u.'s in cell parameters are only used when they are defined by crystal symmetry. An approximate (isotropic) treatment of cell s.u.'s is used for estimating s.u.'s involving l.s. planes.
Refinement. Refinement of *F*^2^ against ALL reflections. The weighted *R*-factor *wR* and goodness of fit *S* are based on *F*^2^, conventional *R*-factors *R* are based on *F*, with *F* set to zero for negative *F*^2^. The threshold expression of *F*^2^ > σ(*F*^2^) is used only for calculating *R*-factors(gt) *etc*. and is not relevant to the choice of reflections for refinement. *R*-factors based on *F*^2^ are statistically about twice as large as those based on *F*, and *R*- factors based on ALL data will be even larger.

### Fractional atomic coordinates and isotropic or equivalent isotropic displacement parameters (Å^2^)

**Table:**

	*x*	*y*	*z*	*U*_iso_*/*U*_eq_	
O1	0.85371 (10)	0.60855 (5)	0.50402 (13)	0.0228 (3)	
O2	0.73858 (10)	0.55038 (5)	0.55581 (14)	0.0244 (3)	
H2O	0.7228 (17)	0.5752 (6)	0.608 (2)	0.037*	
O3	1.04527 (12)	0.41493 (6)	0.13302 (18)	0.0414 (4)	
O4	0.95094 (12)	0.35836 (5)	0.21412 (17)	0.0382 (4)	
O5	0.60266 (10)	0.23665 (5)	0.43762 (14)	0.0236 (3)	
O6	0.67258 (11)	0.29895 (5)	0.32451 (14)	0.0264 (3)	
H6O	0.7045 (17)	0.2740 (6)	0.294 (2)	0.040*	
O7	0.39904 (11)	0.43347 (5)	0.78791 (14)	0.0292 (3)	
O8	0.48451 (11)	0.49026 (5)	0.69235 (14)	0.0268 (3)	
N1	0.75301 (12)	0.69124 (6)	0.65279 (16)	0.0184 (3)	
H1N	0.7837 (14)	0.6684 (6)	0.6080 (19)	0.022*	
N2	0.67877 (11)	0.61741 (5)	0.71728 (15)	0.0179 (3)	
N3	0.63472 (12)	0.69905 (5)	0.80552 (15)	0.0182 (3)	
N4	0.74841 (12)	0.15764 (6)	0.34898 (16)	0.0206 (3)	
H4N	0.7060 (13)	0.1808 (6)	0.375 (2)	0.025*	
N5	0.78776 (12)	0.22892 (6)	0.23078 (16)	0.0195 (3)	
N6	0.87374 (12)	0.14807 (6)	0.20705 (16)	0.0208 (3)	
N7	0.97993 (13)	0.40311 (6)	0.20136 (18)	0.0272 (4)	
N8	0.45857 (12)	0.44542 (6)	0.71057 (15)	0.0196 (3)	
C1	0.68704 (14)	0.66985 (7)	0.72861 (18)	0.0168 (4)	
C2	0.62103 (14)	0.59339 (7)	0.79624 (19)	0.0201 (4)	
H2	0.6140	0.5567	0.7900	0.024*	
C3	0.57137 (14)	0.61991 (7)	0.88640 (19)	0.0206 (4)	
H3	0.5336	0.6025	0.9466	0.025*	
C4	0.57942 (14)	0.67334 (7)	0.88454 (18)	0.0191 (4)	
H4	0.5435	0.6928	0.9429	0.023*	
C5	0.78214 (14)	0.74334 (7)	0.63821 (18)	0.0173 (4)	
C6	0.85205 (14)	0.75203 (7)	0.54896 (19)	0.0191 (4)	
H6	0.8780	0.7234	0.5047	0.023*	
C7	0.88375 (14)	0.80179 (7)	0.52446 (19)	0.0213 (4)	
H7	0.9305	0.8072	0.4625	0.026*	
C8	0.84778 (15)	0.84381 (7)	0.58981 (19)	0.0212 (4)	
H8	0.8695	0.8780	0.5731	0.025*	
C9	0.77973 (15)	0.83529 (7)	0.67983 (19)	0.0214 (4)	
H9	0.7554	0.8640	0.7257	0.026*	
C10	0.74631 (15)	0.78552 (7)	0.70434 (19)	0.0202 (4)	
H10	0.6992	0.7803	0.7660	0.024*	
C11	0.80609 (14)	0.17769 (7)	0.25915 (18)	0.0183 (4)	
C12	0.84094 (14)	0.25086 (7)	0.14286 (19)	0.0211 (4)	
H12	0.8299	0.2867	0.1210	0.025*	
C13	0.91108 (15)	0.22362 (7)	0.0826 (2)	0.0233 (4)	
H13	0.9477	0.2395	0.0186	0.028*	
C14	0.92555 (14)	0.17199 (7)	0.11999 (19)	0.0214 (4)	
H14	0.9750	0.1524	0.0818	0.026*	
C15	0.74319 (14)	0.10683 (7)	0.40058 (19)	0.0187 (4)	
C16	0.68064 (15)	0.10025 (7)	0.50034 (19)	0.0222 (4)	
H16	0.6465	0.1294	0.5308	0.027*	
C17	0.66824 (15)	0.05151 (7)	0.5550 (2)	0.0233 (4)	
H17	0.6251	0.0474	0.6222	0.028*	
C18	0.71820 (15)	0.00886 (7)	0.51246 (19)	0.0228 (4)	
H18	0.7098	−0.0246	0.5500	0.027*	
C19	0.78049 (15)	0.01546 (7)	0.4146 (2)	0.0253 (4)	
H19	0.8151	−0.0138	0.3854	0.030*	
C20	0.79362 (15)	0.06385 (7)	0.3581 (2)	0.0228 (4)	
H20	0.8368	0.0676	0.2909	0.027*	
C28	0.61670 (14)	0.28223 (7)	0.41411 (18)	0.0192 (4)	
C29	0.57437 (14)	0.32553 (7)	0.48937 (18)	0.0180 (4)	
C30	0.59992 (14)	0.37699 (7)	0.46832 (19)	0.0196 (4)	
H30	0.6429	0.3850	0.4032	0.023*	
C31	0.56314 (14)	0.41665 (7)	0.54154 (19)	0.0196 (4)	
H31	0.5808	0.4518	0.5282	0.023*	
C32	0.50015 (14)	0.40359 (7)	0.63449 (18)	0.0177 (4)	
C33	0.47428 (15)	0.35275 (7)	0.65914 (19)	0.0209 (4)	
H33	0.4316	0.3449	0.7249	0.025*	
C34	0.51214 (14)	0.31377 (7)	0.58564 (19)	0.0213 (4)	
H34	0.4955	0.2786	0.6010	0.026*	
C21	0.81529 (14)	0.56496 (7)	0.49520 (18)	0.0183 (4)	
C22	0.85442 (14)	0.52222 (7)	0.41378 (18)	0.0183 (4)	
C23	0.82458 (14)	0.47097 (7)	0.42781 (19)	0.0207 (4)	
H23	0.7765	0.4629	0.4862	0.025*	
C24	0.86457 (15)	0.43168 (7)	0.35713 (19)	0.0217 (4)	
H24	0.8444	0.3966	0.3659	0.026*	
C25	0.93451 (14)	0.44478 (7)	0.27357 (19)	0.0204 (4)	
C26	0.96440 (15)	0.49535 (7)	0.2557 (2)	0.0227 (4)	
H26	1.0115	0.5033	0.1958	0.027*	
C27	0.92389 (14)	0.53422 (7)	0.32744 (19)	0.0198 (4)	
H27	0.9437	0.5693	0.3175	0.024*	

### Atomic displacement parameters (Å^2^)

**Table:**

	*U*^11^	*U*^22^	*U*^33^	*U*^12^	*U*^13^	*U*^23^
O1	0.0274 (7)	0.0153 (7)	0.0280 (7)	−0.0011 (5)	0.0110 (6)	−0.0024 (5)
O2	0.0285 (7)	0.0200 (7)	0.0293 (7)	−0.0027 (6)	0.0161 (6)	−0.0074 (6)
O3	0.0402 (9)	0.0332 (9)	0.0613 (10)	−0.0049 (7)	0.0326 (8)	−0.0181 (8)
O4	0.0361 (9)	0.0197 (8)	0.0628 (10)	−0.0052 (6)	0.0197 (8)	−0.0163 (7)
O5	0.0260 (7)	0.0173 (7)	0.0293 (7)	0.0011 (5)	0.0105 (6)	−0.0013 (5)
O6	0.0329 (8)	0.0208 (7)	0.0309 (7)	0.0078 (6)	0.0181 (6)	0.0019 (6)
O7	0.0396 (8)	0.0246 (7)	0.0313 (7)	−0.0007 (6)	0.0238 (7)	−0.0013 (6)
O8	0.0359 (8)	0.0134 (7)	0.0336 (8)	−0.0042 (6)	0.0136 (6)	−0.0028 (6)
N1	0.0219 (8)	0.0140 (8)	0.0229 (8)	0.0011 (6)	0.0120 (7)	−0.0021 (6)
N2	0.0186 (7)	0.0150 (7)	0.0209 (7)	0.0002 (6)	0.0061 (6)	−0.0003 (6)
N3	0.0188 (8)	0.0173 (8)	0.0195 (7)	0.0015 (6)	0.0068 (6)	−0.0011 (6)
N4	0.0210 (8)	0.0176 (8)	0.0266 (8)	0.0034 (6)	0.0122 (7)	0.0006 (6)
N5	0.0206 (8)	0.0167 (8)	0.0217 (8)	−0.0009 (6)	0.0063 (6)	0.0000 (6)
N6	0.0184 (8)	0.0213 (8)	0.0246 (8)	0.0013 (6)	0.0087 (7)	0.0000 (6)
N7	0.0209 (8)	0.0244 (9)	0.0369 (10)	−0.0021 (7)	0.0082 (7)	−0.0117 (7)
N8	0.0230 (8)	0.0172 (8)	0.0191 (8)	−0.0007 (6)	0.0058 (7)	−0.0013 (6)
C1	0.0159 (8)	0.0175 (9)	0.0167 (8)	0.0007 (7)	0.0033 (7)	0.0007 (7)
C2	0.0191 (9)	0.0184 (9)	0.0227 (9)	−0.0005 (7)	0.0051 (8)	0.0009 (7)
C3	0.0206 (9)	0.0216 (9)	0.0211 (9)	−0.0002 (8)	0.0081 (8)	0.0028 (7)
C4	0.0191 (9)	0.0206 (9)	0.0189 (9)	0.0009 (7)	0.0072 (7)	−0.0002 (7)
C5	0.0177 (9)	0.0159 (9)	0.0180 (9)	−0.0004 (7)	0.0041 (7)	0.0006 (7)
C6	0.0195 (9)	0.0186 (9)	0.0207 (9)	0.0020 (7)	0.0076 (8)	−0.0001 (7)
C7	0.0195 (9)	0.0226 (10)	0.0234 (9)	−0.0009 (8)	0.0085 (8)	0.0024 (8)
C8	0.0219 (9)	0.0173 (9)	0.0232 (9)	−0.0030 (8)	0.0035 (8)	0.0019 (7)
C9	0.0261 (10)	0.0172 (9)	0.0211 (9)	0.0008 (8)	0.0061 (8)	−0.0024 (7)
C10	0.0226 (9)	0.0196 (9)	0.0199 (9)	0.0004 (8)	0.0083 (8)	0.0006 (7)
C11	0.0176 (9)	0.0188 (9)	0.0180 (9)	−0.0022 (7)	0.0034 (7)	−0.0015 (7)
C12	0.0201 (9)	0.0191 (9)	0.0237 (9)	−0.0036 (8)	0.0044 (8)	0.0000 (7)
C13	0.0205 (9)	0.0231 (10)	0.0279 (10)	−0.0032 (8)	0.0090 (8)	0.0015 (8)
C14	0.0179 (9)	0.0251 (10)	0.0226 (9)	−0.0014 (8)	0.0075 (8)	−0.0023 (8)
C15	0.0166 (9)	0.0190 (9)	0.0196 (9)	−0.0014 (7)	0.0030 (7)	0.0005 (7)
C16	0.0213 (9)	0.0232 (10)	0.0234 (9)	0.0026 (8)	0.0082 (8)	−0.0003 (8)
C17	0.0212 (9)	0.0274 (10)	0.0222 (9)	−0.0010 (8)	0.0074 (8)	0.0034 (8)
C18	0.0222 (10)	0.0213 (10)	0.0240 (9)	−0.0042 (8)	0.0038 (8)	0.0026 (8)
C19	0.0270 (10)	0.0205 (10)	0.0296 (10)	0.0003 (8)	0.0091 (9)	−0.0012 (8)
C20	0.0234 (10)	0.0222 (10)	0.0258 (10)	−0.0018 (8)	0.0115 (8)	−0.0006 (8)
C28	0.0182 (9)	0.0203 (10)	0.0188 (9)	0.0038 (7)	0.0037 (7)	0.0016 (7)
C29	0.0174 (9)	0.0180 (9)	0.0174 (8)	0.0012 (7)	0.0022 (7)	0.0005 (7)
C30	0.0182 (9)	0.0213 (9)	0.0201 (9)	0.0006 (7)	0.0064 (7)	0.0009 (7)
C31	0.0207 (9)	0.0157 (9)	0.0223 (9)	−0.0002 (7)	0.0053 (8)	0.0021 (7)
C32	0.0174 (9)	0.0176 (9)	0.0177 (8)	0.0015 (7)	0.0036 (7)	−0.0013 (7)
C33	0.0239 (10)	0.0189 (9)	0.0222 (9)	−0.0025 (8)	0.0101 (8)	0.0003 (7)
C34	0.0232 (9)	0.0159 (9)	0.0253 (9)	0.0000 (8)	0.0065 (8)	0.0015 (7)
C21	0.0183 (9)	0.0198 (9)	0.0163 (8)	0.0030 (7)	0.0034 (7)	0.0013 (7)
C22	0.0169 (9)	0.0177 (9)	0.0181 (8)	0.0021 (7)	0.0002 (7)	−0.0011 (7)
C23	0.0206 (9)	0.0198 (9)	0.0221 (9)	−0.0002 (8)	0.0059 (8)	−0.0007 (7)
C24	0.0211 (9)	0.0177 (9)	0.0250 (9)	0.0004 (8)	0.0034 (8)	−0.0013 (7)
C25	0.0171 (9)	0.0193 (9)	0.0238 (9)	0.0023 (7)	0.0027 (8)	−0.0069 (7)
C26	0.0192 (9)	0.0234 (10)	0.0270 (10)	−0.0031 (8)	0.0085 (8)	−0.0046 (8)
C27	0.0188 (9)	0.0165 (9)	0.0233 (9)	0.0003 (7)	0.0039 (7)	−0.0022 (7)

### Geometric parameters (Å, °)

**Table:**

O1—C21	1.221 (2)	C9—H9	0.9500
O2—C21	1.311 (2)	C10—H10	0.9500
O2—H2O	0.866 (9)	C12—C13	1.372 (3)
O3—N7	1.219 (2)	C12—H12	0.9500
O4—N7	1.227 (2)	C13—C14	1.379 (3)
O5—C28	1.218 (2)	C13—H13	0.9500
O6—C28	1.317 (2)	C14—H14	0.9500
O6—H6O	0.853 (9)	C15—C20	1.392 (2)
O7—N8	1.2243 (18)	C15—C16	1.399 (2)
O8—N8	1.2267 (18)	C16—C17	1.385 (3)
N1—C1	1.357 (2)	C16—H16	0.9500
N1—C5	1.410 (2)	C17—C18	1.382 (3)
N1—H1N	0.876 (9)	C17—H17	0.9500
N2—C2	1.332 (2)	C18—C19	1.381 (2)
N2—C1	1.358 (2)	C18—H18	0.9500
N3—C4	1.332 (2)	C19—C20	1.386 (3)
N3—C1	1.341 (2)	C19—H19	0.9500
N4—C11	1.365 (2)	C20—H20	0.9500
N4—C15	1.407 (2)	C28—C29	1.500 (2)
N4—H4N	0.881 (9)	C29—C34	1.391 (2)
N5—C12	1.331 (2)	C29—C30	1.393 (2)
N5—C11	1.357 (2)	C30—C31	1.386 (2)
N6—C14	1.336 (2)	C30—H30	0.9500
N6—C11	1.338 (2)	C31—C32	1.380 (2)
N7—C25	1.472 (2)	C31—H31	0.9500
N8—C32	1.472 (2)	C32—C33	1.386 (2)
C2—C3	1.373 (2)	C33—C34	1.381 (2)
C2—H2	0.9500	C33—H33	0.9500
C3—C4	1.382 (2)	C34—H34	0.9500
C3—H3	0.9500	C21—C22	1.505 (2)
C4—H4	0.9500	C22—C27	1.389 (2)
C5—C10	1.391 (2)	C22—C23	1.391 (2)
C5—C6	1.398 (2)	C23—C24	1.384 (2)
C6—C7	1.382 (2)	C23—H23	0.9500
C6—H6	0.9500	C24—C25	1.380 (2)
C7—C8	1.385 (2)	C24—H24	0.9500
C7—H7	0.9500	C25—C26	1.381 (3)
C8—C9	1.384 (2)	C26—C27	1.385 (2)
C8—H8	0.9500	C26—H26	0.9500
C9—C10	1.390 (2)	C27—H27	0.9500
			
C21—O2—H2O	110.5 (14)	C20—C15—C16	119.11 (16)
C28—O6—H6O	111.2 (15)	C20—C15—N4	124.96 (15)
C1—N1—C5	130.82 (15)	C16—C15—N4	115.93 (15)
C1—N1—H1N	113.6 (12)	C17—C16—C15	120.40 (16)
C5—N1—H1N	115.5 (12)	C17—C16—H16	119.8
C2—N2—C1	117.41 (15)	C15—C16—H16	119.8
C4—N3—C1	116.00 (15)	C18—C17—C16	120.40 (16)
C11—N4—C15	130.81 (15)	C18—C17—H17	119.8
C11—N4—H4N	112.5 (13)	C16—C17—H17	119.8
C15—N4—H4N	116.6 (13)	C19—C18—C17	119.15 (17)
C12—N5—C11	116.77 (15)	C19—C18—H18	120.4
C14—N6—C11	115.72 (15)	C17—C18—H18	120.4
O3—N7—O4	123.48 (16)	C18—C19—C20	121.40 (17)
O3—N7—C25	118.09 (15)	C18—C19—H19	119.3
O4—N7—C25	118.42 (15)	C20—C19—H19	119.3
O7—N8—O8	123.56 (14)	C19—C20—C15	119.55 (16)
O7—N8—C32	117.95 (14)	C19—C20—H20	120.2
O8—N8—C32	118.48 (14)	C15—C20—H20	120.2
N3—C1—N1	121.65 (16)	O5—C28—O6	124.38 (16)
N3—C1—N2	124.28 (15)	O5—C28—C29	122.83 (15)
N1—C1—N2	114.06 (15)	O6—C28—C29	112.76 (15)
N2—C2—C3	122.12 (17)	C34—C29—C30	119.75 (16)
N2—C2—H2	118.9	C34—C29—C28	119.25 (15)
C3—C2—H2	118.9	C30—C29—C28	120.96 (15)
C2—C3—C4	116.16 (16)	C31—C30—C29	120.58 (16)
C2—C3—H3	121.9	C31—C30—H30	119.7
C4—C3—H3	121.9	C29—C30—H30	119.7
N3—C4—C3	123.71 (16)	C32—C31—C30	118.07 (16)
N3—C4—H4	118.1	C32—C31—H31	121.0
C3—C4—H4	118.1	C30—C31—H31	121.0
C10—C5—C6	118.98 (16)	C31—C32—C33	122.79 (16)
C10—C5—N1	125.10 (15)	C31—C32—N8	118.63 (15)
C6—C5—N1	115.92 (15)	C33—C32—N8	118.57 (14)
C7—C6—C5	120.68 (16)	C34—C33—C32	118.24 (16)
C7—C6—H6	119.7	C34—C33—H33	120.9
C5—C6—H6	119.7	C32—C33—H33	120.9
C6—C7—C8	120.36 (16)	C33—C34—C29	120.54 (16)
C6—C7—H7	119.8	C33—C34—H34	119.7
C8—C7—H7	119.8	C29—C34—H34	119.7
C9—C8—C7	119.11 (16)	O1—C21—O2	124.39 (16)
C9—C8—H8	120.4	O1—C21—C22	122.06 (15)
C7—C8—H8	120.4	O2—C21—C22	113.54 (15)
C8—C9—C10	121.17 (16)	C27—C22—C23	119.97 (16)
C8—C9—H9	119.4	C27—C22—C21	119.21 (16)
C10—C9—H9	119.4	C23—C22—C21	120.78 (15)
C9—C10—C5	119.69 (16)	C24—C23—C22	120.34 (16)
C9—C10—H10	120.2	C24—C23—H23	119.8
C5—C10—H10	120.2	C22—C23—H23	119.8
N6—C11—N5	125.17 (15)	C25—C24—C23	118.30 (17)
N6—C11—N4	121.16 (16)	C25—C24—H24	120.8
N5—C11—N4	113.68 (15)	C23—C24—H24	120.8
N5—C12—C13	122.43 (17)	C24—C25—C26	122.76 (16)
N5—C12—H12	118.8	C24—C25—N7	118.70 (16)
C13—C12—H12	118.8	C26—C25—N7	118.54 (16)
C12—C13—C14	116.37 (16)	C25—C26—C27	118.24 (16)
C12—C13—H13	121.8	C25—C26—H26	120.9
C14—C13—H13	121.8	C27—C26—H26	120.9
N6—C14—C13	123.53 (16)	C26—C27—C22	120.37 (17)
N6—C14—H14	118.2	C26—C27—H27	119.8
C13—C14—H14	118.2	C22—C27—H27	119.8
			
C4—N3—C1—N1	−174.73 (16)	C16—C15—C20—C19	0.4 (3)
C4—N3—C1—N2	6.1 (3)	N4—C15—C20—C19	−178.62 (18)
C5—N1—C1—N3	1.1 (3)	O5—C28—C29—C34	−3.0 (3)
C5—N1—C1—N2	−179.67 (17)	O6—C28—C29—C34	178.90 (16)
C2—N2—C1—N3	−5.1 (3)	O5—C28—C29—C30	174.54 (17)
C2—N2—C1—N1	175.71 (15)	O6—C28—C29—C30	−3.6 (2)
C1—N2—C2—C3	−0.1 (3)	C34—C29—C30—C31	−0.5 (3)
N2—C2—C3—C4	3.6 (3)	C28—C29—C30—C31	−178.06 (16)
C1—N3—C4—C3	−2.2 (3)	C29—C30—C31—C32	−0.5 (3)
C2—C3—C4—N3	−2.4 (3)	C30—C31—C32—C33	1.2 (3)
C1—N1—C5—C10	0.3 (3)	C30—C31—C32—N8	−178.68 (15)
C1—N1—C5—C6	−179.26 (17)	O7—N8—C32—C31	176.93 (16)
C10—C5—C6—C7	−1.1 (3)	O8—N8—C32—C31	−2.5 (2)
N1—C5—C6—C7	178.48 (16)	O7—N8—C32—C33	−3.0 (2)
C5—C6—C7—C8	0.9 (3)	O8—N8—C32—C33	177.59 (16)
C6—C7—C8—C9	0.0 (3)	C31—C32—C33—C34	−0.9 (3)
C7—C8—C9—C10	−0.6 (3)	N8—C32—C33—C34	178.98 (16)
C8—C9—C10—C5	0.4 (3)	C32—C33—C34—C29	−0.1 (3)
C6—C5—C10—C9	0.5 (3)	C30—C29—C34—C33	0.8 (3)
N1—C5—C10—C9	−179.09 (17)	C28—C29—C34—C33	178.42 (17)
C14—N6—C11—N5	−0.4 (3)	O1—C21—C22—C27	−10.3 (3)
C14—N6—C11—N4	179.87 (16)	O2—C21—C22—C27	170.65 (16)
C12—N5—C11—N6	0.7 (3)	O1—C21—C22—C23	167.62 (17)
C12—N5—C11—N4	−179.51 (16)	O2—C21—C22—C23	−11.4 (2)
C15—N4—C11—N6	−1.8 (3)	C27—C22—C23—C24	0.6 (3)
C15—N4—C11—N5	178.41 (17)	C21—C22—C23—C24	−177.32 (17)
C11—N5—C12—C13	0.1 (3)	C22—C23—C24—C25	0.2 (3)
N5—C12—C13—C14	−1.2 (3)	C23—C24—C25—C26	−1.2 (3)
C11—N6—C14—C13	−0.8 (3)	C23—C24—C25—N7	178.25 (16)
C12—C13—C14—N6	1.5 (3)	O3—N7—C25—C24	−176.83 (18)
C11—N4—C15—C20	−5.3 (3)	O4—N7—C25—C24	2.5 (3)
C11—N4—C15—C16	175.72 (18)	O3—N7—C25—C26	2.7 (3)
C20—C15—C16—C17	−0.6 (3)	O4—N7—C25—C26	−178.00 (18)
N4—C15—C16—C17	178.45 (17)	C24—C25—C26—C27	1.4 (3)
C15—C16—C17—C18	0.5 (3)	N7—C25—C26—C27	−178.10 (16)
C16—C17—C18—C19	−0.1 (3)	C25—C26—C27—C22	−0.5 (3)
C17—C18—C19—C20	−0.2 (3)	C23—C22—C27—C26	−0.4 (3)
C18—C19—C20—C15	0.0 (3)	C21—C22—C27—C26	177.51 (16)

### Hydrogen-bond geometry (Å, °)

**Table:**

*D*—H···*A*	*D*—H	H···*A*	*D*···*A*	*D*—H···*A*
O2—H2o···N2	0.866 (9)	1.698 (10)	2.5581 (18)	171 (2)
O6—H6o···N5	0.853 (9)	1.777 (10)	2.6230 (18)	171 (2)
N1—H1n···O1	0.876 (9)	2.147 (10)	3.0217 (18)	176.3 (17)
N4—H4n···O5	0.881 (9)	2.138 (10)	3.0190 (19)	179.6 (19)
C2—H2···O8	0.95	2.41	3.201 (2)	141
C12—H12···O4	0.95	2.43	3.106 (2)	128
C18—H18···O7^i^	0.95	2.56	3.330 (2)	138
C19—H19···O3^ii^	0.95	2.60	3.518 (2)	163
C31—H31···O8^iii^	0.95	2.55	3.238 (2)	129

Symmetry codes: (i) −*x*+1, *y*−1/2, −*z*+3/2; (ii) −*x*+2, *y*−1/2, −*z*+1/2; (iii) −*x*+1, −*y*+1, −*z*+1.
